# The outcome of severely injured patients following traumatic brain injury is affected by gender—A retrospective, multicenter, matched-pair analysis utilizing data of the TraumaRegister DGU^®^

**DOI:** 10.3389/fnins.2022.974519

**Published:** 2022-10-19

**Authors:** Olivia Mair, Frederik Greve, Rolf Lefering, Peter Biberthaler, Marc Hanschen, the TraumaRegister DGU

**Affiliations:** ^1^Department of Trauma Surgery, School of Medicine, Klinikum Rechts der Isar, Technical University of Munich, Munich, Germany; ^2^Faculty of Health, IFOM—Institute for Research in Operative Medicine, University Witten/Herdecke, Cologne, Germany; ^3^Committee on Emergency Medicine, Intensive Care, Trauma Management (Sektion NIS) of the German Trauma Society, Cologne, Germany

**Keywords:** traumatic brain injury, female steroid hormones, estradiol, mortality, matched-pair analysis

## Abstract

**Introduction:**

Traumatic brain injury (TBI) causes a major health-concern globally. Gender-dependent differences in mortality outcome after TBI have been controversially discussed.

**Materials and methods:**

We conducted a retrospective, multicenter, matched-pair analysis using data collected by the TraumaRegister DGU^®^ of the German Trauma Society between 2009 and 2020. All patients after severe trauma with the leading injury of TBI (AIS ≥ 3), above 18 years of age were included. Thereby, 42,034 cases were identified. We used 12 different matching criteria to ensure highly accurate matching and were able to match 11,738 pairs of one female and one male patient.

**Results:**

Average age at injury was 67.5 ± 19.6 years in women and 66.7 ± 19.1 years in men. Mean Injury Severity Score (ISS) was 21.3 ± 8.1 in women and 21.6 ± 8.2 in men. While women were more likely to die within the first week after trauma, the mortality was significantly higher in men overall (30.8 vs. 29.2%, *p* < 0.002). Women were less likely to suffer from multi organ failure (MOF) (27.5 vs. 33.0%) or sepsis (4.5 vs. 7.1%). When comparing younger (≤ 45-years) and older (> 45 years) patients, overall mortality was lower in men (13.1% men vs. 13.4% women) in the younger age group, but in the older group mortality was lower in women (33.8% men vs. 31.8% women).

**Discussion:**

Gender-specific differences in the clinical outcome of severely injured patients with leading TBI could be detected. While women are overall characterized by an advantage in survival, this feature is not equally reproducible in premenopausal women. Therefore, the exact pathophysiological reasons for the described survival advantages of women will have to be explored in further prospective clinical studies.

## Introduction

Until the beginning of the new century sexual dimorphism was not regarded relevant in the medical setting. Yet in recent years, awareness of gender-specific differences has increased steadily. This awareness paves the way to individualized medicine, further acknowledging the naturally given different needs of men and women in diagnostics and treatment after severe trauma (Schoeneberg et al., 2013; Bosch et al., 2018).

Traumatic brain injury (TBI) poses a major health concern globally and is the leading cause of trauma-related death and disability in the younger population (Maas et al., 2017). With an estimated incidence of 287.2 per 100,000 persons it is accompanied by massive socioeconomic costs and consequently deserves special attention in medical research (Majdan et al., 2016).

Primarily, TBI develops in the immediate moment of impact to the brain and presents as micro-/macroscopic bleeding, edema or diffuse swelling of the brain (Weniger et al., 2016). These injuries then trigger the secondary phase of TBI, a phase of apoptosis and necrosis of brain cells, which can develop within minutes to months after the impact. It is caused by disruption of the brain-blood barrier, allowing for accumulation of leukocytes around the injury site, followed by the release of free radicals, a burst in proinflammatory cytokines (i.e., TNF-α, TGF-β, IL-1 and IL-6) and dysregulation of neurotransmitters (Morganti-Kossmann et al., 2007; Weniger et al., 2016).

Gender-specific differences in this secondary phase of TBI, namely in the response of the immune system and inflammatory process, have been extensively investigated. Nevertheless, contradictory findings are presented in literature.

Some authors suggest that women have a worse outcome after TBI than men, in terms of mortality, Glasgow Outcome Scale (GOS) and patient reported outcome measures (PROMs) (Munivenkatappa et al., 2016; Mikolic et al., 2021). On the other hand, multiple studies reported that women suffer from less complications after TBI and generally present better overall outcomes and outperform men after TBI in general. This is often explained by the neuroprotective effect of female steroid hormones, especially estradiol, its derivatives and progesterone (Shahrokhi et al., 2010; Kim et al., 2015, 2017; Weniger et al., 2016). Estrogen and progesterone reduce levels of proinflammatory cytokines, such as IL-1, IL-6, TNF-α and TGF-β, which are mainly responsible for developing brain edema and elevating the intracranial pressure (ICP) (Frink et al., 2007; Shahrokhi et al., 2010; Sarkaki et al., 2013; Mörs et al., 2016; Weniger et al., 2016). Taking it a step further, Kim and coworkers were able to demonstrate lower ICP and less brain edema in rodents after treatment with estrogen sulfate following TBI (Kim et al., 2015, 2017). On the other hand, two independent prospective, placebo-controlled, clinical trials did not find any differences in outcome and mortality when treating patients after TBI with progesterone (Skolnick et al., 2014; Wright et al., 2014). Nevertheless, the exact pathophysiological mechanisms on a cellular level are still not fully understood.

According to the theory of female steroid hormones having a protective effect following trauma, one would expect to find better survival and better outcome especially in women in the reproductive age. Yet, studies showed inconsistent and inconclusive results, which somewhat weakens the validity of the hormone theory (Trentzsch et al., 2015; Ma et al., 2019). Additionally, microglia, resident macrophages in the brain, have been discussed to play a role in causing gender-specific different outcomes after TBI. Their activity also seems to be influenced by sex hormones, yet those exact pathways are not very well understood in terms of TBI (Ma et al., 2019). The contradictory and inconclusive findings in gender-specific differences after TBI warrant more thorough investigations using larger study populations.

Taken together, the aim of this study was to investigate gender-specific differences in the clinical outcome of severely injured patients with TBI as the leading injury. In order to maximize comparability between groups, we conducted a detailed, registry-based, matched-pair analysis. Comparing 11,738 pairs of male and female patients, matched based on similar age, health status and with similar severity of TBI, women seem to have a better survival as compared to men overall. In addition, differences could be detected in the complications following TBI and the overall outcome, the female sex is associated with advantageous outcomes in both categories. Our data, based on a matched-pair analysis including 11,738 pairs, adds to a better understanding of the host’s response to trauma. The current data underlines the need to consider gender as a critical factor in tailoring individualized care in trauma patients, the contradictory data in the literature renders the topic highly relevant for further studies.

## Materials and methods

### Data collection

Data was obtained from the TraumaRegister DGU^®^.

The TraumaRegister DGU^®^ of the German Trauma Society (Deutsche Gesellschaft für Unfallchirurgie, DGU) was founded in 1993. The aim of this multi-center database is a pseudonymized and standardized documentation of severely injured patients. Data are collected prospectively in four consecutive time phases from the site of the accident until discharge from hospital: (A) Pre-hospital phase, (B) Emergency room and initial surgery, (C) Intensive care unit and (D) Discharge. The documentation includes detailed information on demographics, injury pattern, comorbidities, pre- and in-hospital management, course on intensive care unit, relevant laboratory findings including data on transfusion and outcome of each individual. The inclusion criterion is admission to hospital via emergency room with subsequent ICU/ICM care or reach the hospital with vital signs and die before admission to ICU. The infrastructure for documentation, data management, and data analysis is provided by AUC—Academy for Trauma Surgery (AUC-Akademie der Unfallchirurgie GmbH), a company affiliated to the German Trauma Society. The scientific leadership is provided by the Committee on Emergency Medicine, Intensive Care and Trauma Management (Sektion NIS) of the German Trauma Society. The participating hospitals submit their data pseudonymized into a central database via a web-based application. Scientific data analysis is approved according to a peer review procedure laid down in the publication guideline of TraumaRegister DGU^®^. The participating hospitals are primarily located in Germany (90%), but a rising number of hospitals of other countries contribute data as well (at the moment from Austria, Belgium, China, Finland, Luxembourg, Slovenia, Switzerland, The Netherlands, and the United Arab Emirates). Currently, more than 35,000 cases from almost 700 hospitals are entered into the database per year. Participation in TraumaRegister DGU^®^ is voluntary. For hospitals associated with TraumaNetzwerk DGU^®^, however, the entry of at least a basic data set is obligatory for reasons of quality assurance.

The present study is in line with the publication guidelines of the TraumaRegister DGU^®^ and registered as TR-DGU project ID 2021-022. Patient information was double pseudonymized and deidentified prior to analysis. Data in the TraumaRegister DGU^®^ are pseudonymized and routinely collected clinical data obtained from the patients’ chart. Patient consent is obtained prior to entering patient’s data into the TraumaRegister DGU^®^, data collection without patient consent is not possible. This has been waived by the approving ethics committee of the medical faculty of Technical University Munich (TUM), Germany (Project number 48/22 S-NP).

### Patient population

For this study we used data entered between 2009 and 2020. We only used data from German speaking countries (Germany, Austria, Switzerland) to ensure comparability of treatment. TBIs were identified using the abbreviated injury scale (AIS) codes from the AAAM Manual. The AIS code book includes over 2000 different injuries, with each injury receiving a severity classification of 1 (minor) to 6 (not survivable). Only patients with a severe TBI (AIS head ≥ 3) were included (Gennarelli and Wodzin, 2006).

Organ failure was defined as a SOFA-score ≥ 3 for each organ using the SOFA-score classification. Multi organ failure (MOF) was defined as organ failure in two or more organs on at least two consecutive days (Vincent et al., 1996).

Low-energy trauma was defined as low falls (< 3 m), as opposed to high-energy trauma such as motor-vehicle accidents, falls from great heights, etc.

As the aim of this study was to investigate the gender specific differences after TBI in severely injured patients, we only included patients with a severe TBI (AIS of 3 or higher). In addition, to ensure that the TBI was the leading and most severe injury, only patients with concomitant injuries with a severity of AIS 3 or less were included.

We excluded pregnant women, patients < 18 years of age at the time of the accident and patients, where essential information for our study, namely the sex, Glasgow Coma Scale (GCS)-Score and/or blood pressure at the scene, were missing. In addition, all patients who were transferred to the reporting hospital or who were transferred out after initial treatment were excluded as key information was missing in these cases.

A total number of 42,034 cases were thereby identified and used for matching.

### Matching

To make the matching process as precise as possible, we used 12 different matching criteria (Table 1) to pair one woman with one man, who matched in every different category. These 12 variables were chosen in such a way that the patients could be compared as accurately as possible in terms of their health status before the trauma, their age and, in particular, their exact injury pattern.

**TABLE 1:** Matching criteria used to match the 42,034 cases included in this study.

Matching criteria	Specification/ grouping
Age group (years)	18–25/26–35/36–45/46–55/56–65/66–75, > 75
Severity of TBI (AIS)	3/4/5/6
Concomitant abdominal injury (AIS)	0–1/2–3
Concomitant thoracic injury (AIS)	0–1/2–3
Concomitant injury of the spine (AIS)	0–1/2–3
Concomitant pelvic injury (AIS)	0–1/2–3
Concomitant injury of the extremities (AIS)	0–1/2–3
ASA score before injury	1–2/3–4
Prehospital GCS	3–8/9–13/14–15
Prehospital shock	Systolic BP ≤ 90/ > 90 mmHg
Trauma mechanism	Low/high energy trauma
Type of documentation	Reduced/standard form

AIS, Abbreviated injury scale; TBI, traumatic brain injury; GCS, Glasgow Coma Scale; BP, blood pressure; ASA, American Society of Anesthesiologists.

Shock was defined as hypotension with systolic blood pressure ≤ 90 mmHg. Low impact trauma included low falls from < 3 m.

ASA scores were divided into 2 groups, the healthy population with ASA scores of 1 and 2, and the pre-diseased population with ASA scores of 3 and 4.

We chose not to use ISS as a separate matching criterion, as it can be rather inaccurate in reflecting a patient’s exact injury pattern. We chose to use a more detailed approach to represent each patient’s injury pattern by using the AIS scores in all 6 different body regions utilized to calculate the ISS.

Additionally, we matched patients by age groups, by prehospital GCS (also grouped) and by extended vs. basic data set in order to ensure comparability of entered data.

Applying these matching criteria 11,738 pairs of one female and one male patient, who matched in every category, could be found.

### Statistical analysis

Data was analyzed using the Statistical Package for the Social Sciences (SPSS, version 24; IBM Inc., Chicago, IL, USA). Patients’ characteristics were described using mean and standard deviation (SD) for continuous variables. Absolute numbers and relative percentages were used for categorical variables. Significances were calculated using paired *t*-tests or Fisher’s-Exact Test, where appropriate. *P*-values < 0.05 were considered statistically significant.

Due to the large number of patients, even minor differences became formally significant. Interpretation thus should focus on clinical relevance rather than on formal significance.

GraphPad Prism Version 9.2.0 (GraphPad Software, LLC.) was used to generate graphs.

When comparing female and male patients, values for the female subgroup will always be named first.

The Revised Injury Severity Classification II (RISC II) score is a commonly used scoring systems developed using data from the TraumaRegister DGU^®^, which helps in predicting outcome after multiple trauma. Thirteen variables are used to calculate the RISC II score, including, but not limited to sex, age, preexisting injuries, GCS scores, pupil reaction, trauma mechanism and shock index (Lefering et al., 2014).

## Results

### Patient characteristics

According to the above-mentioned criteria, we were able to identify 11,738 pairs of patients who matched in all categories.

Table 2 summarizes patient characteristics when compared between men and women.

**TABLE 2:** Patient characteristics and specific data comparing men and women.

		Women *n* = 11,738	Men *n* = 11,738
		
		Mean ± SD	Mean ± SD
Age (years)		67.5 ± 19.6	66.7 ± 19.1
ISS		21.3 ± 8.1	21.6 ± 8.2
Trauma mechanism	Traffic accident	31.3%	26.9%
	High fall (≥3 m)	8.0%	11.9%
	Low fall (<3 m)	52.8%	52.4%
	Penetrating trauma	1.1%	2.3%
	Suicide	1.5%	2.3%
Isolated TBI (AIS ≥ 3)		55.2%	54.0%
Pre-hospital (phase A)			
		**Median (IQR)**	**Median (IQR)**
GCS on scene		11 (6–14)	11 (5–14)
Shock on scene (BP ≤ 90 mmHg)		4.7%	4,4%
Resuscitation (CPR) on scene		2.4%	2.5%
Intubation on scene		37.9%	37.5%
Emergency room/Initial surgery (phase B)			
Whole-body CT		67.7%	70.3%
Shock (systolic BP ≤ 90 mmHg)		5.0%	5.1%
Blood transfusion		4.6%	4.1%
Coagulopathy (INR ≥ 1.4, or PTT ≥ 40, or Quick’s value ≤ 60)		16.1%	18.4%
Acidosis (base excess < –6.0)		10.6%	11.7%
Intensive care unit/hospital (phase C)			
ICU admission		93.9%	95.0%
		**Median (IQR)**	**Median (IQR)**
Days on ICU		3 (1–8)	3 (1–11)
Days in hospital		10 (4–18)	10 (4–19)

The average age at injury was 67.5 ± 19.6 years in women and 66.7 ± 19.1 years in men. Mean ISS was at 21.3 ± 8.1 and 21.6 ± 8.2 in women and men, respectively. Detailed review of the trauma mechanism showed that women were more likely to have suffered from traffic accidents (31.3 vs. 26.9%, *p* < 0.001), but were significantly less likely to suffer from penetrating trauma (1.1 vs. 2.3%, *p* < 0.001) and high falls from above 3 meters (8.0 vs. 11.9%, *p* < 0.001). Additionally, over half of our study population suffered from a low fall (< 3 m; 52.8 vs. 52.4%).

Over half of our study population sustained an isolated TBI in the female and male (55.2 vs. 54.0%) subgroups alike.

In the prehospital phase (Phase A) women and men seem to have similar characteristics in terms of shock, GCS, resuscitation and intubation on scene.

In the trauma room phase (Phase B) less women received whole body computed tomography (67.7 vs. 70.3%) and were also less likely to present acidosis (10.6 vs. 11.7%) and coagulopathy (16.1 vs. 18.4%).

In the hospital phase (Phase C) women were less likely to be submitted to the intensive care unit (ICU) (93.9 vs. 95.0%) and overall had shorter stays in ICU (6.7 ± 9.3 days vs. 8.3 ± 11.0 days) and in hospital (13.4 ± 14.5 days vs. 14.4 ± 15.5 days) after the trauma.

### Outcome

As explained before, the RISC II score is used to predict mortality in patients after multiple trauma. Female sex is a known beneficial factor for survival according to the RISC II score, so it is not unexpected that the RISC II score was higher in men than in women (Lefering et al., 2014). Expected mortality based on RISC II score was 26.3% in men and 24.9% in women, while in our study population overall hospital mortality was even higher with 30.8% in men and 29.2% in women.

Overall, the outcome after TBI was beneficial in women (Table 3). Women were significantly less likely to have MOF (27.5 vs. 33.0%, *p* < 0.001) and sepsis (4.5 vs. 7.1%, *p* < 0.001). When each organ system is considered separately, the differences in the rates of cardiac (26.9 vs. 31.5%, *p* < 0.001), respiratory (14.8 vs. 18.3%, *p* < 0.001) and cerebral organ failure (38.6 vs. 42.9%, *p* < 0.001) are particularly striking.

**TABLE 3:** Outcome after TBI in multiple trauma patients with individual organ outcome.

	Women (*n* = 11,738)	Men (*n* = 11,738)	*P*-value
Sepsis	4.5%	7.1%	*p* < 0.001
Organ failure…			
… liver	0.8%	1.0%	*p* = 0.134
… kidney	2.6%	3.3%	*p* = 0.014
… coagulopathy	7.8%	9.3%	*p* = 0.004
… lung	14.8%	18.3%	*p* < 0.001
… heart	26.9%	31.5%	*p* < 0.001
… brain	38.6%	42.9%	*p* < 0.001
Multi organ failure	27.5%	33.0%	*p* < 0.001
Discharged home	33.7%	31.8%	*p* = 0.002
Expected mortality based on RISC II	24.9%	26.3%	*p* < 0.001
Overall hospital mortality	29.2%	30.8%	*p* = 0.005

RISC, Revised injury severity classification; SD, standard deviation.

Furthermore, women were more often discharged home (31.8 vs. 33.7%), which could also serve as a quantifier of the better clinical outcome in women.

#### Mortality

**FIGURE 1:**
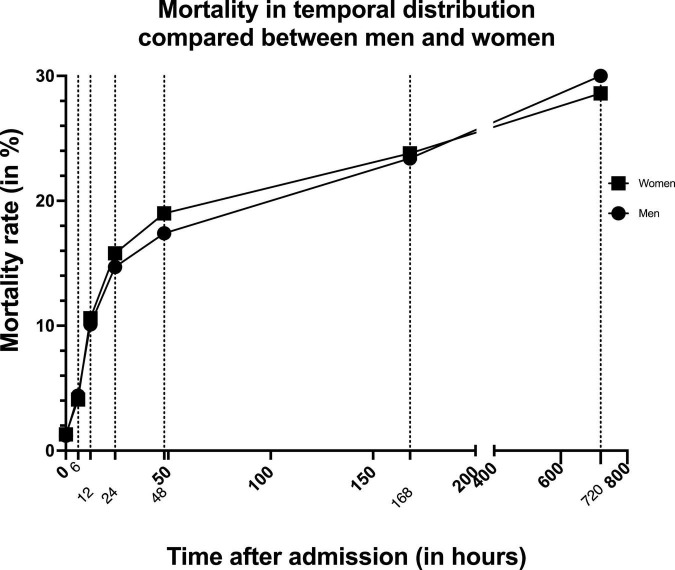
Graph showing mortality rate in temporal distribution compared between men and women. Dotted lines indicate the different measurement times.

If we take a closer look at the time of death in the temporal distribution, we notice that women die more frequently than men within the first week after trauma (Table 4). After approximately 1 week this negative effect reverses and women become more likely to survive (Figure 1). From there on this trend is confirmed with women being more likely to survive at 30 days after trauma (28.6 vs. 30.0%, *p* < 0.014). In addition, the overall mortality rate is significantly lower in women than in men (29.2 vs. 30.8%, *p* = 0.002).

**TABLE 4:** Mortality rates shown in temporal distribution in the overall study population.

	Mortality rate (%) (f/m)	*P*-value
Cumulative mortality …		
… In trauma room	1.3/1.2	*p* = 0.406
… Within 6 h	4.1/4.4	*p* = 0.364
… Within 12 h	10.6/10.1	*p* = 0.304
… Within 24 h	15.8/14.7	*p* = 0.014
… Within 48 h	19.0/17.4	*p* = 0.002
… Within 7 d	23.8/23.4	*p* = 0.415
… Within 30 d	28.6/30.0	*p* = 0.014
Overall hospital mortality	29.2/30.8	*p* = 0.005

H, hours; d, days; m, male; f, female.

**FIGURE 2:**
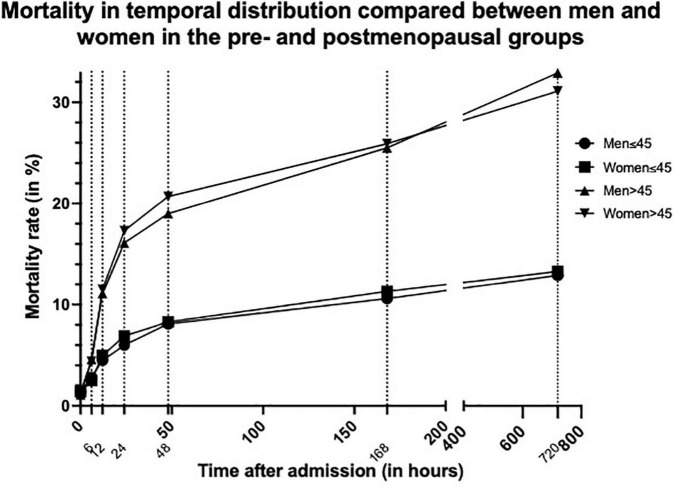
Graph showing mortality rate in temporal distribution compared between men and women in the pre (≤45 years)- and postmenopausal (>45 years) groups. Dotted lines indicate the different measurement times.

In order to serve the theory of hormonal differences providing benefits following TBI for women, we compared the younger and older study population (≤ 45-/ > 45-years-old) (Figure 2). This cut-off was chosen to depict the approximate age at which women go into menopause. While the temporal distribution of mortality in the older age-group is similar to that of the overall group, in the younger age-group this effect is not as clear (Table 5). The overall mortality is lower in older women than in older men (31.8 vs. 33.8%, *p* < 0.003). Nevertheless, the overall mortality of younger women is even a little bit higher than in men of the same age group, even though this is not statistically significant (13.4 vs. 13.1%, *p* = 0.839).

**TABLE 5:** Mortality rates shown in temporal distribution in the younger (≤45-years old) vs. the older population (>45-years old).

Mortality rates f/m (in %)	≤45-years old *n* = 1,692/1,692	*P*-value	>45-years old *n* = 10,046/10,046	*P*-value
Died…				
… In trauma room	1.4/1.4	p = 1.010	1.3/1.1	*p* = 0.346
… Within 6 h	2.5/2.8	*p* = 0.671	4.4/4.6	*p* = 0.454
… Within 12 h	5.0/4.5	*p* = 0.571	11.5/11.1	*p* = 0.373
… Within 24 h	6.9/6.0	*p* = 0.328	17.3/16.1	*p* = 0.022
… Within 48 h	8.3/8.1	*p* = 0.851	20.7/19.0	*p* = 0.002
… Within 7 days	11.3/10.6	*p* = 0.546	25.9/25.5	*p* = 0.519
… Within 30 days	13.3/12.9	*p* = 0.760	31.1/32.9	*p* = 0.007
Hospital mortality	13.4/13.1	*p* = 0.839	31.8/33.8	*p* = 0.003

H, hours; d, days; m, male; f, female.

## Discussion

To the best of our knowledge this is by far the largest matched-pair analysis investigating gender-specific differences in patients with leading TBI after severe trauma.

This study shows that the overall mortality of severely injured women with leading TBI is significantly lower as compared to men (29.2 vs. 30.8%; *p* < 0.005).

This is in contrast to the findings by Schoeneberg and coworkers, who found in their cohort study of multiply injured patients, that females had a higher overall mortality rate following multiple trauma as compared to men. Even when conducting a matched-pair analysis they found women at a higher risk of death following trauma induced injury (Schoeneberg et al., 2013).

It might therefore be reasonable to assume that TBI as the leading injury is an important factor to be considered when predicting mortality following multiple trauma.

As explained above, there are several different theories as to why women have a better outcome after TBI than men. Whether it be the influence of genes, hormones, or a combination of the above is still under debate. The theory of female steroid hormones being one of the most important beneficiary factors in the outcome after TBI has been well investigated especially in laboratory models, yet clinical application of this theory is not as concise (Shahrokhi et al., 2010; Trentzsch et al., 2015; Weniger et al., 2016; Kim et al., 2017; Ma et al., 2019). This could for one be due to the fact that men are significantly overrepresented in most cohort studies as they are more prone to trauma in general (Mikolic et al., 2021). On the other hand mostly male rats have been used especially in older rodent model studies, therefore neglecting other gender specific differences unique to the female system (Ma et al., 2019).

When discussing the possible beneficiary effects of female steroid hormones on TBI, one should also be aware, that hormone levels can easily be manipulated by many factors. For one large numbers of women take exogenous hormones, i.e., oral contraceptives, hormonal supplements in postmenopausal women or in terms of cancer therapy, which significantly changes baseline hormonal levels. Furthermore, hormone levels vary significantly throughout the menstruous cycle (Berry et al., 2009; Mikolic et al., 2021). There is extremely limited information in terms of clinical prospective studies investigating hormone levels and/or exogenous hormonal supplements at the time of trauma in the clinical setting (Wagner et al., 2011; Ma et al., 2019).

This might be another reason why hormonal theories have mostly been proven in laboratory settings, where hormone levels are always in natural occurrence and are not manipulated (Duncan and Garijo-Garde, 2021).

In order to investigate the hormonal theory further we separated pre- and postmenopausal women. We determined the cut-off of the premenopausal and postmenopausal women to be at 45 years of age, as an estimated time of menopause in industrialized countries. Nevertheless, this only serves as a very general estimate and does in no way represent reality as age at natural menopause is influenced by many factors, including ethnicity, socioeconomic status and parity and is individual in every woman (Gold, 2011).

In the younger group of patients under the age of 45, the previously described survival advantage of women could not be demonstrated in our study (Table 5). The overall mortality was marginally higher in younger women compared to younger men (13.4 vs. 13.1%). Nevertheless, while this difference in mortality of 0.3% is statistically significant, one is inclined to question the clinical relevance of this very slight difference. Also, the temporal distribution did not follow the same pattern as described in the overall study population.

Opposed to this, older women seem to have the previously described survival advantage, when compared to men of the same age groups (33.8 vs. 31.8%) (Table 5). These similarities could for one be explained by the much larger number of matched-pairs in the older age group (10,046 matched-pairs > 45- years old vs. 1,692 matched-pairs ≤ 45- years old).

Berry et al. (2009) presented similar findings in their retrospective cohort study, which analyzed sex-differences after moderate to severe head trauma. They stated that peri- (46–55 years) and postmenopausal (> 55 years) women showed better survival compared to men of the same age groups, while premenopausal (< 46 years) women did not have this same advantage. Trentzsch and coworkers also found contradictory results in their matched- pair analysis of severely injured patients. While this study did not put any focus on TBI in particular, they found postmenopausal (> 45 years) women to have lower rates of sepsis and better survival, while premenopausal (< 45 years) women were less likely to contract MOF than men of the same age-groups (Trentzsch et al., 2015).

When focusing specifically on the temporal distribution of deaths in our study population, women were more likely to die within the first week after trauma than men. Yet this trend reversed after approximately 1 week (Figure 1), making women more likely to survive overall. This can be attested to the fact that women were significantly less likely to contract MOF (27.5 vs. 33.0%, *p* < 0.0001) and presented lower sepsis rates (4.5 vs. 7.1%, *p* < 0.001) in this study. This is in accordance with findings of several previous studies. Similar to the protective effect on TBI, female steroid hormones have been discussed to have a beneficial effect after trauma hemorrhage and development of systemic inflammatory response syndrome (SIRS) and sepsis by lowering levels of proinflammatory cytokines, leading to lower overall mortality rates after major trauma in women (Choudhry et al., 2006; Frink et al., 2007; Schoeneberg et al., 2013; Trentzsch et al., 2014; Weniger et al., 2016; Bosch et al., 2018).

The mean age at trauma in our study population was 67.1 ± 19.3. This is significantly higher compared to the average age at trauma of 54.2 years reported by the TraumaRegister DGU^®^ in 2020 (Deutsche Gesellschaft für Unfallchirurgie [DGU] et al., 2021). This difference can be explained by our inclusion process, where we included also patients with an isolated TBI. This pattern of trauma is increasingly found in the elderly population, often resulting from falls from low heights, i.e., standing positions or stairs (Maas et al., 2017). Consequently, this explains the high rate of the injury mechanism “falls from low height,” which makes up more than half of our study group.

Nevertheless, as we used each trauma mechanism and age as criteria in the matching process, we do not expect bias due to misrepresentation of data.

While to the best of our knowledge this is by far the largest matched-pair analysis analyzing gender-specific differences in the outcome of patients following major trauma with leading TBI, there are some limitations to this study.

This is a registry-based study. Even though the inferiority of data in registry-based studies due to faulty or missing data entry is well known, it allows for large study populations and therefore highly powered investigations. By excluding patients with missing essential data, we believe that this extremely large data mass will even out most of the known potential inaccuracies.

The matching process in a matched-pair analysis is always accompanied by some inaccuracies. Nevertheless, by using 12 different matching criteria we believe we reached quite a precise level of comparability between each patient. We included matching criteria in many different aspects, from demographic data to trauma mechanism and injury patterns and therefore believe to have matched patients with high accuracy. Furthermore, we tried to use categorial variables and category groups as it is much more robust to fault, while losing some detail.

Another limitation results from the fact that the hormonal status of the patients at the time of trauma is not known. This lack in data and knowledge has been much discussed in literature and should further be investigated more extensively in future prospective trials (Berry et al., 2009; Ma et al., 2019).

In this study we only distinguished between male and female patients, as the new category of “diverse” was only implemented in the V2020 entry form of the TraumaRegister DGU^®^. The number of cases entered are therefore not yet statistically significant. While we believe that our very large study population would not be influenced significantly by the rather small population identifying as trans- or cisgender, the effect of TBI on the transgender community should be considered in future studies (Deutsche Gesellschaft für Unfallchirurgie [DGU] et al., 2021; Duncan and Garijo-Garde, 2021).

## Conclusion

This study corroborates the presence of gender-specific differences in the outcome of severely injured patients following TBI. Overall, when compared to the opposite gender at similar age, health status and with similar severity of TBI, women seem to have better probability of survival than men. Furthermore, women suffer from less complications after TBI and therefore have better outcome overall. Yet, this effect is not recognized as clearly in premenopausal women under the age of 45, which calls into question the theory that female steroid hormones are the main reason for the survival advantage in women after severe injury.

More studies will be needed in the future to examine the reasons for these gender differences in more detail. Prospective clinical trials are necessary, which record the baseline hormonal levels of all participants at the time of trauma in order to validate hormonal theories in the clinical settings.

## Data availability statement

The data is collected by the TraumaRegister DGU^®^ and can be made available after internal review of the request. Requests to access the datasets should be directed to support@auc-online.de.

## Author contributions

OM contributed to conception and design of the study and wrote the first draft of the manuscript. FG and PB edited and corrected the manuscript. PB supervised the manuscript writing. RL performed the statistical analysis. MH contributed to conception and design of the study, edited the manuscript, and supervised the manuscript writing. TraumaRegister DGU collected the data. All authors contributed to manuscript revision, read, and approved the submitted version.
